# Thromboembolism in Patients with Cancer: A Practical Guide to Recurrent Events

**DOI:** 10.31083/j.rcm2511406

**Published:** 2024-11-19

**Authors:** Sergey Kozhukhov, Nataliia Dovganych

**Affiliations:** ^1^Clinical Pharmacology & Cardio-Oncology Department, NSC “The M.D.Strazhesko Institute of Cardiology”, 03680 Kyiv, Ukraine; ^2^Cardio-Oncology Center, 03680 Kyiv, Ukraine

**Keywords:** cancer, VTE, recurrent VTE, VTE progression, treatment

## Abstract

Cancer patients have an increased risk of venous thromboembolism (VTE), and VTE is the second most common cause of death among them. Anticoagulation plays a key role in the treatment of cancer-associated thrombosis (CAT). Low-molecular-weight heparin (LMWH) or direct oral anticoagulants (DOACs) are effective and generally safe options for cancer-associated VTE. However, those patients have a 10–20% risk of VTE recurrence in spite of using anticoagulants. The main reasons for recurrent VTE (rVTE) can be non-compliance, inadequate dosing of anticoagulants, thrombocytopenia and malignancy progression. Despite the publication of major guidelines regarding the management of CAT, the treatment of patients with rVTE is undefined. Treatment options for rVTE include bridging to LMWH in cases of oral anticoagulants use, LMWH dose escalation, and sometimes considering inserting a vena cava filter. This review paper summarizes the management of cancer-associated VTE, risk factors for rVTE and the treatment algorithm of rVTE.

## 1. Introduction

Cancer patients have an increased risk of venous thromboembolism (VTE) compared to the general population, and VTE is the second leading cause of death after cancer itself [1]. Anticoagulant therapy is the key option for the treatment and prophylaxis of VTE [2, 3, 4, 5, 6, 7]. A combination of cancer and thrombosis may encounter higher rates of recurrent VTE (rVTE) and major bleeding than those without malignancy. Current treatment options of cancer-associated thrombosis (CAT) include low-molecular-weight heparin (LMWH), direct oral anticoagulants (DOACs), unfractionated heparin, fondaparinux, and vitamin K antagonists (VKAs). Deciding on a treatment strategy, selection of anticoagulants, duration of treatment, and adjustment of regimens in special situations are the major problems in CAT management. The goals of the present article are (i) to analyze treatment options how to manage these patients, (ii) to discuss challenges in cases of progressive and recurrent VTE in real clinical practice, and (iii) to propose an individual treatment algorithm in special situations.

## 2. Management of Cancer-associated VTE 

The current standard of care issued by the 2021 National Comprehensive Cancer Network guidelines [3], the 2021 American Society of Hematology guidelines [4], the 2022 International Initiative on Thrombosis and Cancer (ITAC) guidelines [5], the 2023 American Society of Clinical Oncology guidelines Guideline Update [6] and the 2023 ESMO Guidelines [7] recommend using LMWH, DOACs, unfractionated heparin, fondaparinux, and VKAs for the management of CAT during 6 months based on evidence from randomized clinical trials (RCTs). To date, LMWH and DOACs are the most frequently used pharmacological anticoagulant agents for CAT.

## 3. Current Status of Cancer-associated VTE Treatment

### 3.1 VKA 

The evidence on the treatment of CAT with VKAs is based on RCTs conducted in patients without cancer [8]. LMWHs were assigned for 5–10 days followed by the VKA international normalized ratio (INR) target of 2.0 to 3.0 [9]. It is not easy for the VKAs to keep the INR in range due to interactions with chemotherapy drugs that affect VKA metabolism, inconsistent dietary intake due to anorexia, nausea or vomiting, low body mass, and low albumin [10]. Therefore, the therapy of choice with LMWH or DOAC (for 6 months) should be preferred over VKAs.

According ESMO guidelines, if rVTE occurs in patients on VKAs with an INR in the subtherapeutic range, then they should be treated with LMWH until the INR reaches its therapeutic values again [11]. If rVTE occurs on VKAs in the therapeutic range, a switch to LMWH is recommended by most guidelines [2, 3, 4, 5, 6, 7], without a subsequent return to the VKA. According to ESMO recommendations, if rVTE occurs in patients taking VKAs with an INR in the sub-therapeutic range, they should be treated with LMWH until the INR reaches therapeutic values.

### 3.2 LMWH

LMWHs have remained the first-line treatment for VTE for more than two decades [12, 13, 14, 15, 16]. Among the five RCTs only the CLOT [13] trial reported statistically improved rates of rVTE with dalteparin compared with a VKA and the other four (CANTHANOX, ONCENOX, LITE, and CATCH [12, 13, 14, 15, 16]) demonstrated non-statistically significant reductions in rVTE with an LMWH compared with a VKA.

### 3.3 DOACs

The current guidelines recommend DOAC (apixaban or rivaroxaban) or LMWH as the initial treatment (5 to 10 days) for patients with CAT [3, 4, 5, 6, 7].

For the short-term treatment of CAT (up to 6 months), the current guidelines indicate DOAC (apixaban, edoxaban, or rivaroxaban) over LMWH.

For the long-term treatment (>6 months) for patients with malignancy and VTE, guidelines suggest using DOACs or LMWHs [2, 3, 4, 5, 6].

Four recent RCTs have compared the efficacy and safety of DOACs vs. LMWH in cancer patients. The Hokusai cancer study compared edoxaban with subcutaneous dalteparin during a 12-month treatment in cancer patients with acute VTE. rVTE occurred in 7.9% in the edoxaban group and in 11.3% in the dalteparin group at the 6- and 12 month follow-up points [17].

Select-D was a pilot open-label trial in patients with VTE, comparing rivaroxaban and dalteparin [18]. The incidence of rVTE over 6 months was significantly lower in the rivaroxaban than in the dalteparin arm (4% vs. 11%).

In both studies [17, 18], DOACs showed non-inferiority in reducing rVTE compared with LMWH however they were associated with a higher risk for major bleeding complications in patients with gastrointestinal (GI) cancer.

In an open-label RCT - the ADAM VTE trial - apixaban and dalteparin were compared in patients with CAT [19]. rVTE occurred in 0.7% in the apixaban group in comparison to 6.3% in the dalteparin, group. rVTE was 0.7% in the apixaban group in comparison to 6.3% in the dalteparin group.

Major bleeding events up to 6 months occurred in 0% of patients assigned to apixaban in contrast to 1.4% of patients on dalteparin. The composite bleeding endpoint (major or clinically relevant non-major bleeding) was 6% for each arm.

In the largest RCT, Caravaggio study, rVTE at 6 months of follow-up occurred in 5.6% of patients randomized to apixaban compared with 7.9% of those assigned to dalteparin [20]. Major bleeding occurred in 0.6% of patients in the apixaban arm and in 1.8% in the conventional-therapy group.

In the ADAM VTE and CARAVAGGIO studies, there was no excess of GI bleeding in patients treated with apixaban, including those with GI cancer.

The CASTA DIVA study, which explored 3 months of treatment with rivaroxaban, found a rate of rVTE of 6.4%, compared to 10.1% in patients who received dalteparin. The study was stopped prematurely and was unable to confirm noninferiority against dalteparin for the prevention of rVTE [21].

A meta-analysis of four studies (Hokusai cancer, Select-D, ADAM VTE, and CARAVAGGIO) evaluating the efficacy and safety of DOACs compared with LMWH for CAT demonstrated that DOACs significantly decrease recurrent thrombosis compared with dalteparin without significantly increasing major bleeding. Edoxaban significantly increased major bleeding events compared with dalteparin, while rivaroxaban increased clinically relevant non-major bleeding compared with dalteparin and other DOACs [22].

The non-inferior risk of rVTE among patients with active cancer was confirmed in the CANVAS study, where the use of a DOAC compared with an LMWH was without differences in rates of bleeding or death [23].

It should be noted that DOACs increase the risk of bleeding, so in patients with GI cancer and upper or unresected lower GI cancer, LMWH may be preferred.

### 3.4 Vena Cava Filter in CAT Treatment

An inferior vena cava (IVC) filter should be considered in cancer patients with absolute contraindications for anticoagulation therapy such as active bleeding or a high risk of bleeding with recently diagnosed deep vein thrombosis (DVT). Anticoagulation should be started immediately after the resolution of any contraindications. Retrievable filters in comparison to permanent filters are preferable in the cancer setting.

According to American Society of Clinical Oncology (ASCO), an IVC filter may be suggested to patients with absolute contraindications to anticoagulation in the acute setting if the thrombus propagation is considered life-threatening [9].

An IVC filter may be offered in addition to anticoagulation in patients with thrombosis progression despite optimal treatment. As indicated by ITAC, an IVC filter may be considered for initial treatment when anticoagulation is contraindicated or when pulmonary embolism (PE) occurs despite optimal anticoagulation [5].

It is recommended that contraindications to anticoagulation should be periodically reviewed and anticoagulation can be considered.

## 4. Recurrent Cancer-associated VTE and VTE Progression

Recurrent VTE: Venous thrombosis (PE and/or DVT) of a site that was either previously uninvolved or had interval documentation of DVT or PE resolution [24]. The diagnosis of rVTE must be established by comparing current and previous imaging examinations.

The issue of a correct diagnosis of recurrence is clinically relevant because many patients with previous VTE may present with signs or symptoms suggesting the possibility of a recurrent event [25].

VTE progression: New PE and/or DVT episode, occurring or worsening after 30 days of treatment [26].

### 4.1 Rate and Causes of Recurrent Cancer-associated VTE 

Patients with CAT have a high risk of rVTE despite receiving anticoagulation. Risk factors for recurrence are identified in the RIETE registry [27]. Rates of rVTE in cancer patients differ between tumor sites and depend on the type of anticoagulation agents. In patients treated with VKA, recurrence rates were 10 to 17% during the first 6 months after VTE [12, 16]. Rates of recurrence with the LMWH, based on the CLOT trial, were 7–9% [12, 16], and with DOACs varied between RCTs from 3.9 to 7.9% [17, 18, 19, 20].

Other reasons for rVTE may include non-compliance, temporary interruption of treatment due to bleeding or surgery, inappropriate dosing, disease progression, or drug-drug interactions that may reduce the anticoagulant effect.

Based on the data from modern guidelines, in the case of rVTE, the next options should be used: (i) switching to LMWH if a DOAC or VKA is used, (ii) LMWH dose escalation, (iii) or insertion of IVC filter in the setting of rVTE despite optimal anticoagulation or in case of contraindication to anticoagulation due to bleeding.

### 4.2 Non-compliance as a Cause of VTE Recurrence or Progression

Non-compliance is a key reason of “obvious treatment failure”. Compliance with VKAs is easy to monitor with an INR at the time of the rVTE. Controlling noncompliance with the DOAC regimen is even more challenging without available and validated tools. Available literature data supports that non-compliance with DOACs is common [28].

If the patient is not compliant, they should be managed with the same anticoagulant therapeutic strategies and doses as a patient with a first VTE to prevent a subsequent recurrence. In the case of non-compliant VTE events, there is no clear answer as to whether to remain on the current treatment option or switch to another anticoagulant.

No RCT has explored different treatment options in patients with therapeutic failure of anticoagulation, and there are no clear data to guide the management strategy for these patients.

Additionally, there is no published research covering treatment options after DOAC failure—DOAC dose escalation or switching DOAC to another agent?

In cases of rVTE on DOAC, the same approach as with patients who experienced VKA failure can be proposed by switching to a therapeutic dose of LMWH.

### 4.3 Inappropriate Dosing of Anticoagulant as the Cause of VTE Recurrence or VTE Progression

Although underdosing may potentially increase the risk of thromboembolic complications, overdosing may result in bleeding.

Therefore, an appropriate initial dose of anticoagulant is crucial for patients with active malignancies.

Reduced doses of the anticoagulant should be considered only in patients with a high bleeding risk, with thrombocytopenia, and renal failure. Current guidelines recommend treatment with a therapeutic dose of LMWH for platelets (PLT) ≥ 50 < 100 × 10^9^/L, a reduced dose to 50% of LMWH for PLT ≥ 30 < 50 × 10^9^/L, and discontinuation of therapy for PLT <30 × 10^9^/L [7].

In the case of LMWH underdosing, a weight-adjustment dose of LMWH should be prescribed according to guidelines.

In the case of DOAC underdosing, switching DOAC to the therapeutic dose of LMWH can be the best strategy. In case of successful therapy with LMWH, it is possible to switch LWMH back to oral treatment, typically to another DOAC.

### 4.4 Recurrent VTE on Optimal Anticoagulant Treatment 

Patients with rVTE, despite therapeutic doses of anticoagulant therapy, should be assessed for several factors, such as treatment compliance, heparin-induced thrombocytopenia (HIT), antiphospholipid syndrome, thrombophilia (protein C, protein S, Factor V Leiden mutation, and antithrombin deficiency), malignancy compression or invasion resulting in tumor-thrombosis [29]. Management options for recurrent VTE on optimal anticoagulation include an alternative anticoagulant regimen or an increase in LMWH dose.

### 4.5 Recurrent VTE in Cancer Patients on Optimal Dose of LMWH

In a small retrospective study, 47 patients with cancer and rVTE who were already receiving a therapeutic dose of LMWH had a dose escalation of 20 to 25% for 4 weeks [30]. The recurrence rate on super therapeutic dose was 8.6% during 3 months, and the author suggests that escalating the dose of LMWH can be effective for treating patients that are resistant to standard, weight-adjusted doses of LMWH [31]. The international ISTH registry demonstrated no significant difference in the risk of further recurrences over 3 months between patients who had a dose escalation of ≥20–25% and those with a standard dose of LMWH [32]. The ACCP guidelines recommend a dose escalation of LMWH by 25–33% [31], and ASCO recommends a 20 to 25% increase [6].

### 4.6 Recurrent VTE in Patients Receiving a Therapeutic Dose of DOACs

Data of rVTE on DOACs is limited. A meta-analysis of VTE trials (26,872 patients) demonstrated a 2% risk of rVTE during the acute treatment phase [33].

The one-year cumulative rate of rVTE in cancer patients is estimated to be at about 21%, compared to only 7% in patients without cancer [34]. A meta-analysis of 29 studies (8000 patients with CAT), demonstrated the overall risk of rVTE 23.7 events per 100-patient years [35]. In the SELECT-D study, the 6-month risk of rVTE in patients treated with rivaroxaban was 4% vs 11% with LMWH [18], in the Hokusai VTE Cancer trial, risk of rVTE at 6- and 12-months in edoxaban arm was 4.4% and 7.9%, vs 6.7% and 11.3% in LMWH arm, respectively [17], and in the Caravaggio trial, patients assigned to the apixaban arm had a risk of rVTE at 6 months of 5.6% vs 7.9% with LMWH [19]. So, the risk of rVTE in cancer patients varied from study to study from 4 to 5.6%.

The systematic review of four RCTs (Caravaggio, ADAM VTE, SELECT-D, and Hokusai VTE) that analyzed 2894 patients with CAT, demonstrated that treatment with a DOAC significantly decreased rVTE in comparison to dalteparin (5.6% and 9.1%, respectively) without any significant increase in major bleeding event rates (4.8% vs 3.6%) [22].

Three DOACs—apixaban, edoxaban, and rivaroxaban are not only more treatment-effective but also more cost-effective than LMWH [36].

## 5. Clinical Cases

### 5.1 CASE 1: Recurrent VTE on DOAC

**Fig. 1.**
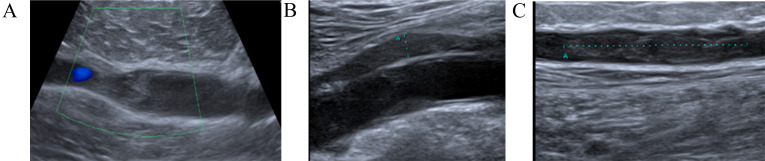
**Dynamics imaging with compression ultrasound (CUS)**. (A) Right lower extremity CUS showing extensive occlusive thrombus in the popliteal vein. (B) After four weeks CUS shows partial recanalization of the right popliteal vein. (C) CUS revealed an extension of the right popliteal deep vein thrombosis (DVT) with a new DVT in the right femoral vein.

A 58-year-old man was diagnosed with sigmoid colon adenocarcinoma and received surgery followed by three courses of chemotherapy (CapeOx). He was referred to the Cardio-Oncology Center and noticed the sudden onset of right calf pain without any previous trauma one week before his visit. His initial physical exam revealed a tender and swollen right calf. Compression ultrasound (CUS) demonstrated DVT of the right popliteal vein (Fig. 1A). He was prescribed rivaroxaban 15 mg twice a day for the first 21 days, then 20 mg once daily; he adhered to this regimen. After four weeks, CUS showed partial recanalization of the right popliteal vein and normal blood flow in the deep veins of the left leg (Fig. 1B). Two weeks later, the patient returned to the Cardio-Oncology Center with progressive swelling in his right leg and right thigh, accompanied by pain. Physical examination revealed a swollen, tender right lower extremity. CUS revealed an extension of the right popliteal DVT with a new DVT in his right thigh (Fig. 1C). Computed tomographic pulmonary angiography (CTPA) did not show PE. Recurrent VTE was diagnosed and DOAC was switched to enoxaparin in a therapeutic dose based on body weight twice a day on the recommendation of a cardio-oncologist. At the 2-month Follow-up (FU), subtotal recanalization in both lower limbs was confirmed by CUS.

#### Learning Points

This case presents the challenges faced when managing rVTE despite adherence to therapeutic anticoagulation. For patients with rVTE during either VKA or DOAC therapy, current guidelines suggest temporary switching to LMWH for at least one month. For patients already receiving LMWH, it is recommended to increase the dose by 25%.

### 5.2 CASE 2: Progressive Thrombosis from DVT to PE on DOAC

**Fig. 2.**
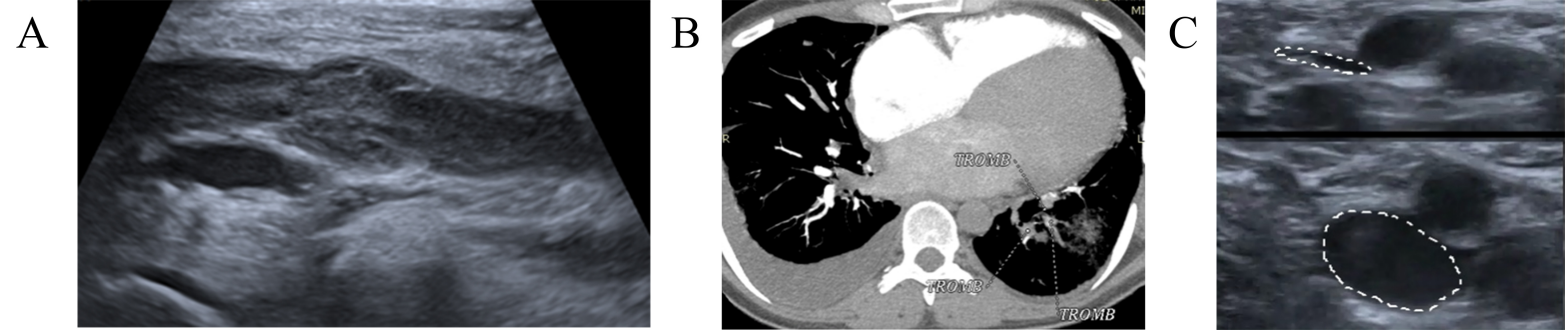
**Dynamics imaging with CUS and computed tomographic pulmonary angiography (CTPA)**. (A) CUS demonstrated DVT of the left popliteal and femoral veins. (B) CTPA showed segmental and sub-segmental thrombi in the right PA. (C) Control CUS detected subtotal recanalization without signs of new thrombosis, normal blood flow in the deep veins of both legs. TROMB, trombus; CUS, compression ultrasonography; CTPA, computed tomography pulmonary angiogram; DVT, deep venous thrombbosis; PA, pulmonary artery.

A 49-year-old woman with breast cancer (ductile, stage IIIA, hormone-dependent, Luminal type B) received radical mastectomy, four cycles of chemotherapy (AC), followed by 12 T (paclitaxel), local radiotherapy, and hormonal therapy with Tamoxifen. The patient was referred to the outpatient Cardio-Oncology department for cardiac function monitoring. During the physical examination the cardio-oncologist paid attention to a slight enlargement of the left leg that otherwise had no other symptoms. A CUS demonstrated DVT of the left popliteal and femoral vein (Fig. 2A). The patient denied any risk factors that could have provoked VTE, since she had neither a history of previous cardiovascular or respiratory disease nor a history of PE or DVT. Therefore, treatment with rivaroxaban 15 mg twice a day was started. Two weeks later patient suddenly developed dyspnea and tachycardia. A CTPA showed segmental and sub-segmental thrombi in the right PA (Fig. 2B). Also, CUS found only a slight thrombi recanalization in the left popliteal and femoral veins. LMWH—enoxaparin in a dose of 1 mg/kg subcutaneously every 12 hours was prescribed. In a 2-month FU, CUS detected subtotal recanalization with normalization of blood flow in the deep veins of the left leg (Fig. 2C). The patient was switched to DOAC therapy with 5 mg of apixaban, twice daily. The patient remained stable up to the 6-month FU.

#### Learning Points

Data about the therapeutic failure of DOAC is limited. Detailed analysis of VTE recurrence is very important for decision-making of future anticoagulation strategies. In this case, we observed clot propagation and VTE progression from DVT to PE. DOAC was switched to LMWH in therapeutic doses twice a day for at least one month. In cases of a good clinical effect, we can return to DOAC therapy, but with another molecule, therefore we switched the patient to apixaban. The total duration of anticoagulant therapy should be at least 6 months from the time of a rVTE event.

### 5.3 CASE 3: DOAC-refractory VTE/DOAC-resistant VTE

**Fig. 3.**
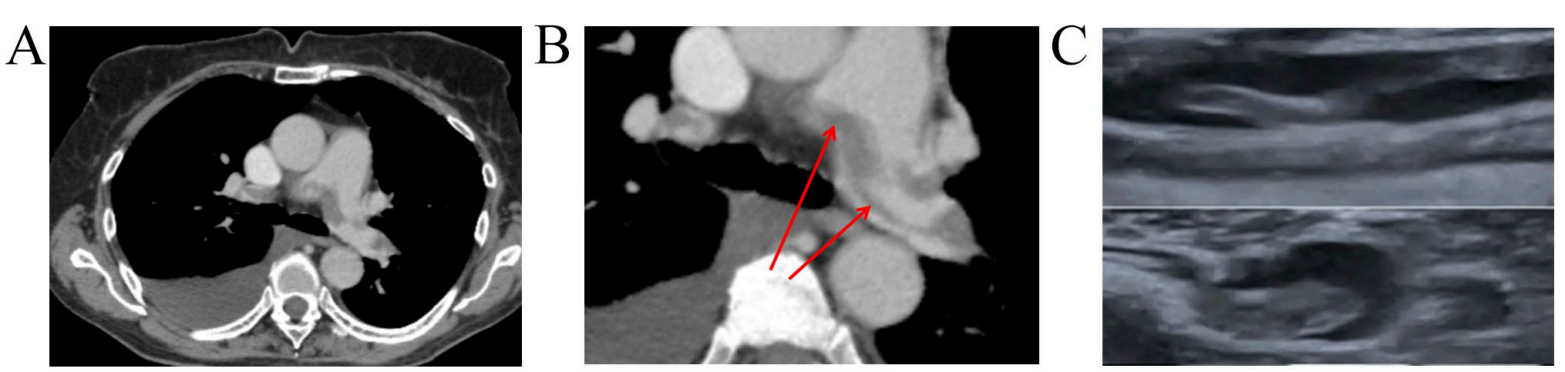
**Dynamics imaging with CUS and CTPA**. (A) CTPA showed a large saddle embolus in the bifurcation of the pulmonary trunk that extends to both PA. (B) CTPA showed non-resolved saddle embolus in PA bifurcation, thrombi in segmental and sub-segmental branches of both PA. (C) Lower-limb CUS showed slight recanalization and floating thrombus in 1 month FU. CUS, compression ultrasonography; CTPA, computed tomography pulmonary angiogram; PA, pulmonary artery; FU, follow-up.

A 71-year-old female, with non-Hodgkin mantle cell lymphoma, stage IVB, with involvement of thoracic, mediastinal, retroperitoneal lymphatic nodes and pleura was referred to Cardio-Oncology Center. Prior to cancer treatment, the risk of VTE was 1 point per Khorana’s score. After the third RB (rituximab+bendamustine) course she demonstrated grade 4 neutropenia, a high temperature of 39 °C, sudden dyspnoea, and weakness. Pneumonia signs and right pleural effusion were detected on X-ray. She received antibacterial therapy and steroids and underwent thoracentesis. However, the patient’s condition continued to deteriorate: she suffered from dyspnea, tachycardia, chest pain, edema of the legs, and heart failure (HF) signs (New York Heart Association, NYHA III). Electrocardiogram showed sinus tachycardia with a heart rate (HR) of 105 bpm. Transthoracic echocardiogram demonstrated a slightly dilated right ventricular with mildly reduced systolic function and left ventricle ejection fraction (EF) was 53%. Troponin I level was normal (0.015 ng/mL), while D-dimer elevated at 11,000 ng/mL. PE was suspected. CTPA showed a large saddle embolus in the bifurcation of the pulmonary trunk, which extends to both lung arteries with embolization up to 60% (Fig. 3A), right-sided pleural effusion, and pneumonia signs. Lower-limbs CUS revealed occlusive thrombotic masses in the deep veins of both legs. PE of intermediate-low risk and DVT were diagnosed.

Anticoagulation with rivaroxaban 15 mg twice a day, antibacterial, and heart failure therapy were prescribed.

After a 1-month patient was referred to the Cardio-Oncology Center for decision-making for restart of cancer treatment. She was asymptomatic, HR 72 bpm, blood pressure (BP) 130/78 mm Hg, EF 57%, D-dimer—1500 ng/mL.

However, CTPA scan demonstrated non-resolved saddle embolus in PA with obstruction of 50%. Lower-limb CUS showed only 10% recanalization and floating thrombus (Fig. 3B).

DOAC-resistant VTE was diagnosed and LMWH (enoxaparin 1 mg/kg subcutaneously every 12 hours) was prescribed. Additionally, we performed laboratory analysis for thrombophilia (protein C, protein S, antithrombin deficiency, and Factor V Leiden mutation), and no abnormalities were found.

She was scheduled for a follow-up examination in one month of the treatment period with enoxaparin. The patient demonstrated no PE signs on computed tomography (CT), and partial (60%) recanalization of DVT by lower-limb CUS. By the Cardio-oncology team’s decision, the patient was switched to another DOAC - apixaban. At the 3-month follow-up subtotal recanalization in both lower limbs was confirmed by CUS (Fig. 3C). The patient remained stable throughout the 6-month FU.

#### Learning Points

In cases of DOAC failures (no recanalization and/or progression of VTE), resistance to this DOAC should be suspected after excluding all possible causes of recurrence and non-effectiveness (non-compliance, genetic (inherited) or acquired thrombophilia, etc.).

In such situations, patients should be switched to a therapeutic dose of LMWH. In cases of successful recanalization, it is possible to re-prescribe a DOAC, however another DOAC type should be used.

## 6. Ukrainian Cardio-oncology Team’s Approach

**Fig. 4.**
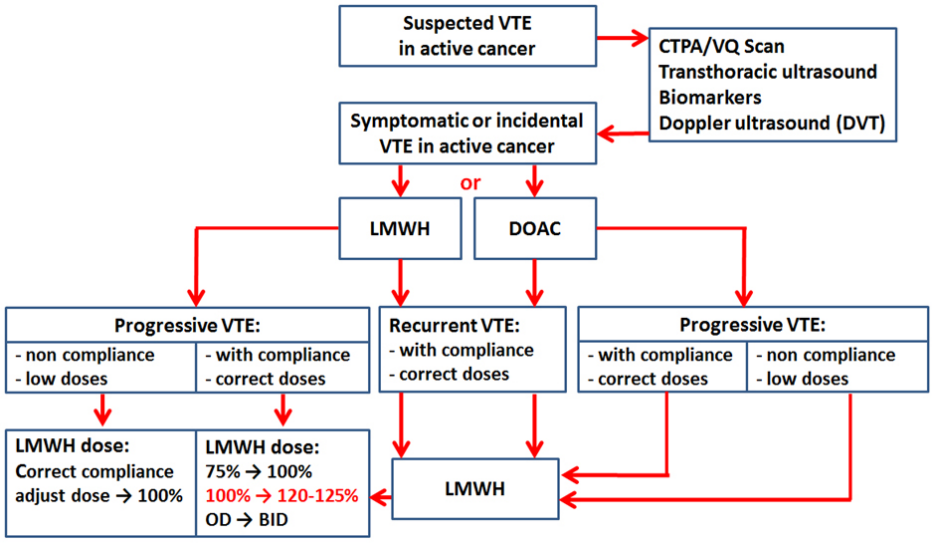
**Venous thromboembolism (VTE) treatment algorithm for anticoagulation in VTE recurrence or progression**. CTPA, computed tomography pulmonary angiogram; VQ, ventilation/perfusion; LMWH, low-molecular-weight heparin; DOAC, direct oral anticoagulants; OD, once a day; BID, bis in die.

Our approach to VTE treatment in cases of rVTE or VTE progression is summarized in Fig. 4. Cancer patients are treated mostly with LMWH or DOACs for 6 months. The decision to start anticoagulant therapy and the choice of the medication in patients with confirmed symptomatic or incidental VTE is made by the Cardio-Oncology team. We have analyzed the profile of patients without the effectiveness of anticoagulant treatment and divided them into thefollowing groups: (i) VTE recurrence or VTE progression on the low dose of anticoagulant; (ii) VTE recurrence on the optimal dose of anticoagulant; (iii) VTE progression on the optimal dose of anticoagulant. A simple algorithm for decision-making of anticoagulation in these patients was proposed (Fig. 4).

DOAC treatment failure or DOAC resistance should be suspected in cases of persistent symptoms of the original DVT/PE, no recanalization by CUS/CT, and/or progression of VTE despite 4 to 6 weeks of optimal anticoagulation after excluding all possible causes of rVTE and non-effectiveness. There are no clear recommendations about their efficacy monitoring, increasing or changing the DOAC dose in case of DOAC failure.

Due to the lack of formal guidelines for the management of these patients, we suggest that switching to LWMH is the best option.

## 7. Discussion

In terms of terminology, a distinction should be made between recurrent VTE and progressive VTE. The term “recurrent VTE” is mostly used as an endpoint in all RCTs. However, in clinical practice, doctors can often face VTE progression. Poor adherence, inadequate dosage, temporary cessation, cancer progression, drug interactions, etc., are the main causes of this complication [37]. VTE recurrence, despite therapeutic anticoagulation, is the most frequent complication and can occur regardless of the type of anticoagulant used [38].

It is essential to investigate and understand the possible causes of rVTE. The first step is to determine whether the patient received a therapeutic dose of anticoagulant or a reduced dose. A reduced dose (below the therapeutic dose) in the absence of other reasons for dose adjustment may be due to the initial low dose or the patient’s non-compliance. If a low dose of LMWH was used, increasing the dose to the therapeutic range is recommended by guidelines. If a therapeutic dose was used, the ACCP guidelines recommend a dose escalation of LMWH by 25 to 33% [32], while ASCO recommends a 20–25% increase in the LMWH dose [6]. However, there are still numerous unmet needs. Practically, it is often difficult to maintain patients on a twice-daily injection regimen for a long time. A question that is to be discussed is the following: if the patient used an optimal dose of LMWH once a day, should the medical specialist increase the dose by 20–25% in case of rVTE, or switch to a twice-daily subcutaneous injection? We suggest that LMWH use twice daily from the start of the treatment is optimal for cancer patients to reduce LMWH concentration fluctuations, and prevent rVTE [6].

Another question is if DOAC is used at a sub-therapeutic dose, will its dose increasing to the standard range be effective? For example, the frequency of inappropriate dosing of DOAC in patients with atrial fibrillation was 15%, most of these patients were under-dosed, and patients were at higher risk of stroke and VTE [39]. To date, there is no clear answer to this question; it is not known whether resistance to the drug will develop when it is switched from a low dose to a therapeutic dose. If VTE recurrence/progression occurs on a low dose of DOAC, these patients should be switched to the therapeutic dose of LMWH. In cases of VTE recurrence/progression on the optimal dose of DOAC, the patient should be transferred to the therapeutic dose of LMWH.

From a clinical point of view, refractoriness is usually defined as breakthrough thrombosis on standard doses of anticoagulant. Suspected new thrombosis or thrombosis extension requires objective confirmation by using appropriate imaging tools. In this regard, thrombophilia must also be mentioned, as constitutional or acquired laboratory abnormalities in coagulation that predispose to VTE or VTE recurrence. Many studies have shown an association between thrombophilia and VTE [4, 29].

HIT is an important risk factor for rVTE in cancer patients. VTE occurs in about 50% of confirmed HIT cases [24]. HIT may cause rVTE due to a combination of cancer and HIT. When HIT is suspected, especially in a cancer patient with unexplained thrombocytopenia, standard methods of diagnosis and treatment should be used [40].

Treatment of patients with CAT and rVTE is not well determined. Thus, some issues are not studied in RCTs, meaning there are no experimental studies, and in these cases, we can only rely on expert opinions.

## 8. Conclusions

In summary, many patients with CAT are at significant risk of rVTE.

Today, according to current recommendations, LMWH and DOACs are the best treatment options for cancer-associated VTE.

Unfortunately, in special clinical situations, the best strategy for managing rVTE during anticoagulation treatment is poorly defined, and the decision may be based on an expert’s opinion - very low-certainty evidence.

In all cases of recurrence or progression of VTE, to guide decision-making that concerns correction of anticoagulant therapy, the potential cause of recurrence should be identified and corrected if possible.

Therefore, research priorities should focus on the identification of patients with a high risk of recurrence/progression of VTE, and additional trials are needed to guide rVTE management.

Thus, Cardio-Oncology consulting and strict monitoring are needed to decide on the best anticoagulated options for these patients.
